# Microdialysis Sampling from Wound Fluids Enables Quantitative Assessment of Cytokines, Proteins, and Metabolites Reveals Bone Defect-Specific Molecular Profiles

**DOI:** 10.1371/journal.pone.0159580

**Published:** 2016-07-21

**Authors:** Yvonne Förster, Johannes R. Schmidt, Dirk K. Wissenbach, Susanne E. M. Pfeiffer, Sven Baumann, Lorenz C. Hofbauer, Martin von Bergen, Stefan Kalkhof, Stefan Rammelt

**Affiliations:** 1 University Center of Orthopedics and Trauma Surgery and Center for Translational Bone, Joint and Soft Tissue Research, University Hospital “Carl Gustav Carus”, TU Dresden, Dresden, Germany; 2 Department of Molecular Systems Biology, Helmholtz-Centre for Environmental Research - UFZ, Leipzig, Germany; 3 Institute of Pharmacy, Faculty of Biosciences, Pharmacy and Psychology, University of Leipzig, Leipzig, Germany; 4 Division of Endocrinology and Metabolic Bone Diseases, Department of Medicine III, University Hospital “Carl Gustav Carus”, TU Dresden, Dresden, Germany; 5 Institute of Biochemistry, Faculty of Biosciences, Pharmacy and Psychology, University of Leipzig, Leipzig, Germany; 6 Centre for Microbial Communities, University of Aalborg, Aalborg East, Denmark; 7 Department of Bioanalytics, University of Applied Sciences and Arts of Coburg, Coburg, Germany; 8 Center for Regenerative Therapies Dresden (CRTD), Dresden, Germany; University of New Mexico HSC, UNITED STATES

## Abstract

Bone healing involves a variety of different cell types and biological processes. Although certain key molecules have been identified, the molecular interactions of the healing progress are not completely understood. Moreover, a clinical routine for predicting the quality of bone healing after a fracture in an early phase is missing. This is mainly due to a lack of techniques to comprehensively screen for cytokines, growth factors and metabolites at their local site of action. Since all soluble molecules of interest are present in the fracture hematoma, its in-depth assessment could reveal potential markers for the monitoring of bone healing. Here, we describe an approach for sampling and quantification of cytokines and metabolites by using microdialysis, combined with solid phase extractions of proteins from wound fluids. By using a control group with an isolated soft tissue wound, we could reveal several bone defect-specific molecular features. In bone defect dialysates the neutrophil chemoattractants CXCL1, CXCL2 and CXCL3 were quantified with either a higher or earlier response compared to dialysate from soft tissue wound. Moreover, by analyzing downstream adaptions of the cells on protein level and focusing on early immune response, several proteins involved in the immune cell migration and activity could be identified to be specific for the bone defect group, e.g. immune modulators, proteases and their corresponding inhibitors. Additionally, the metabolite screening revealed different profiles between the bone defect group and the control group. In summary, we identified potential biomarkers to indicate imbalanced healing progress on all levels of analysis.

## Introduction

Bone exhibits the remarkable ability for self-repair without scarring [1]. The healing process consists of three overlapping stages: (i) inflammation, (ii) repair, and (iii) remodeling. Each stage is orchestrated by a specific set of biological events [2]. Thereby, several key cells like osteoblasts, osteoclasts and osteocytes as well as immune cells interact with each other and their microenvironment. Pro-osteogenic and angiogenic factors but also inflammatory chemokines and cytokines, are secreted and interact with the extracellular matrix (ECM) and several cell types resulting in optimal fracture repair and restoration of the skeletal functions [3–5]. A persistent inflammatory response and a failed transition to the repair stage can disrupt the bone healing process and thus result in delayed, impaired or completely absent bone healing after acute fractures. Thus, the inflammatory stage which is simultaneously activated with the formation of the fracture hematoma may be crucial for consolidation [6, 7]. During the initial stage of inflammation activated platelets and degranulating neutrophils infiltrate the fracture site and release inflammatory cytokines [8]. Although the function of the cells involved in this process is well described, the knowledge about the molecular mechanisms after wounding is still scarce. This includes the temporal and spatial interplay of individual immune mediators as well as the downstream response of the cells in the wound area, in terms of protein expression and metabolic adaptions. To promote this understanding, it is relevant to identify and quantify chemokines and cytokines, other proteins like ECM components, proteases and protease inhibitors as well as various metabolites directly at their site of action.

Since the wound fluid adjacent to the fracture site contains all the soluble factors released into the microenvironment of the fracture hematoma, its assessment provides remarkable insights into the wound milieu [8, 9]. Microdialysis has become an established method to sample wound fluid from a variety of tissues, including skin, brain and liver [10–12]. We recently established microdialysis in a critical-size bone defect and demonstrate that cytokines can be sampled and quantified by enzyme-linked immunosorbent assays (ELISA) continuously over 24 hours [13]. The sampling is based on the diffusion of molecules across a semi-permeable membrane driven by a concentration gradient. However, due to the high biological activity of most cytokines, their concentration in body fluids is rather low [14]. Nevertheless, the direct analysis of the chemokines and cytokines is beneficial because genomic approaches do not display the functional microenvironment at the site of injury. Measuring mRNA or DNA expression may indicate a cellular upregulation of the relevant chemokines and cytokines but transcriptional regulations or posttranslational modifications as well as protein degradation can modulate the protein abundance [15]. A major benefit of microdialysis is the fact that it is minimally invasive and allows continued sampling during the wound healing process and thus a time-dependent analysis of metabolites and cytokines of the same individuals.

Mass spectrometry is an ideal tool for the identification and quantification of proteins and metabolites from body fluids in an untargeted approach and the combination of microdialysis and proteomics has been widely used to identify and quantify proteins and peptides simultaneously [16–18]. However, the sampling of proteins by using microdialysis is highly challenging, because the recovery depends to a great extent on the properties of the proteins including molecular weight, shape, charge of the molecule, binding to the membrane, and a possible degradation of the proteins [12]. Additionally, a high salt content, a high complexity and a low overall concentration of proteins in the wound fluids interfere with successful comprehensive screening. Thus, proteomic analyses were limited to the quantification of only few dozen to about 150 proteins regardless of the sampling technique [17, 19, 20]. Recently, we established a sampling approach that allows the identification of more than 500 proteins by using a solid phase extraction of proteins adsorbed to the microdialysis catheter. Moreover, this approach can be combined with the classical continuous sampling of cytokines and of metabolites [21].

In order to identify bone-specific markers we applied microdialysis in combination with proteomics and metabolomics in two different wound scenarios in a rat bone defect (BD) and soft tissue wound (STW). Dialysates were used for analysis of selected chemokines and cytokines by ELISA to obtain time-resolved data during the first 24 hours after injury.

## Materials and Methods

### Animal Surgery

The study was approved by the Agency for the Inspection of Veterinary and Food Materials of the Government of Saxony (permission no. 24–9168.11-1/2013-31). All animals were housed according to the European guidelines for the care and use of laboratory animals. Forty-four male Wistar rats with an average body weight of 350 g were obtained from Janvier (Le Genest Saint Isle, France) and held in the animal care unit for at least 7 days before the experiments.

The rats were anesthetized with a solution of ketamine (100 mg/kg body weight) and xylazine (10 mg/kg body weight) and kept under anesthesia up to 12 hours (administration of ketamine/xylazine through a permanent intraperitoneal catheter every 90–120 min). After shaving and disinfection of the right hind leg a 3 cm longitudinal incision was made and the femur was surgically exposed by dissecting the thigh muscle. A five-hole plate (Stryker, Hamburg, Germany) was fixed with 4 screws (1.5 x 6 mm) on the femur. In twenty four rats a 5 mm bone defect was created using a wire saw (group: bone defect, BD). In twenty rats the bone was not injured and only a soft tissue wound was created down to the femur (control group: soft tissue wound, STW). The microdialysis catheter (CMA/20, CMA Microdialysis AB, Solna, Sweden, cut-off 100 kDa) was inserted either into the soft tissue defect near the bone or into the bone defect. The muscle was closed with absorbable sutures and the skin was closed with suture clips. The remaining animals were allowed to wake up and move freely after closure of the muscle and skin. After 12 hours these animals were anesthetized again and microdialysis catheter was inserted after opening the wound area 1 cm. The rats were covered with thin sheets to avoid hypothermia. Additionally, a mixture of 0.25 mL 0.9% NaCl solution and 0.25 mL 10% glucose was administered every 120 min through the permanent catheter to avoid hypoglycemia and dehydration. The rats were sacrificed under anesthesia at the end of the experiment by CO_2_ inhalation.

### Microdialysis

Before insertion, the microdialysis catheter was primed with an initial flush (5 μL/min) for 15 min. The membrane was perfused with perfusion fluid (PER, 147 mmol/L NaCl, 4 mmol/L KCl, 2.3 mmol/L CaCl_2_, 1% bovine serum albumin) at a flow rate of 2 μL/min using a CMA402 microdialysis pump (CMA Microdialysis AB, Solna, Sweden). The samples were collected over 12 h in 3 h intervals on ice. To avoid protein degradation PMSF (phenylmethylsulfonylfluoride, final concentration 1 mmol/L) was added to each collection tube. The dialysates were aliquoted and immediately stored at -20°C. The catheter was explanted at the end of the experiment and stored at -20°C.

### Histology

At the end of the experiment the fracture hematoma was explanted, fixed with 4% formaldehyde, embedded in paraffin, and cut into 2 μm-sections for Pappenheim´s staining. For quantification mononucleated and multinucleated cells were counted in 10 randomly distributed squares per section on three subsequent slices per fracture hematoma.

### ELISA

The concentration of 15 cytokines and growth factors were analyzed with commercially available ELISA kits (Table 1). The ELISA assays were performed according the manufacturer’s instructions. Mean and standard deviation were calculated when at least 3 values were above the detection limit of the relevant ELISA.

**Table 1:** List of used enzyme-linked immunosorbent assay (ELISA) kits. All kits were purchased as indicated and used according to the manufacture’s guide.

Cytokine/growth factor	ELISA kit	Company	Detection limit
IL-6	Rat Il-6 Quantikine ELISA kit	R&D Systems, Wiesbaden, Germany	10 pg/mL
TGF-ß1	Mouse/Rat/Porcine/Canine TGF-beta1 Quantikine ELISA kit	R&D Systems, Wiesbaden, Germany	1.7 pg/mL
CXCL1 **(CINC-1)**	Rat CXCL1/CINC-1 Quantikine ELISA kit	R&D Systems, Wiesbaden, Germany	1.1 pg/mL
CXCL2 **(MIP-2)**	MIP-2 ELISA kit	Life Technologies, Darmstadt, Germany	1 pg/mL
CXCL3	Chemokine (CXC motif) Ligand 3 (CXCL3) ELISA kit	Cloud-Clone Corp., Houston, USA	5.5 pg/mL
CXCL4 **(PF4)**	Platelet factor 4 (PF4) ELISA kit	Cloud-Clone Corp., Houston, USA	128 pg/mL
CXCL7	CXCL7 Rat ELISA kit	Abcam, Cambridge, UK	80 pg/mL
SDF-1 **(CXCL12)**	Chemokine (CXC motif) Ligand 12 (CXCL12) ELISA kit	Cloud-Clone Corp., Houston, USA	0.125 ng/mL
IL-10	Rat IL-10 Quantikine ELISA kit	R&D Systems, Wiesbaden, Germany	10 pg/mL

### Sample Preparation and Acquisition for Proteomics

The sample preparation and acquisition was conducted as previously described [21]. Briefly, protein adsorbates (n = 4 for each wound scenario explanted after 12 h) were eluted from the catheter membrane with Laemmli buffer for 5 min at 95°C. The eluted proteins were separated by 1D-SDS-PAGE. The samples were sliced to 8 gel fractions each and an in-gel proteolytic cleavage was conducted using trypsin. 52 ng of the Proteomics Dynamic Range Standard (UPS2, Sigma Aldrich, Seelze, Germany) was spiked into each fraction. For control experiments, proteins from microdialysates were purified using Vivacon spin tubes (10 kDa MWCO, Sartorius, Göttingen, Germany). Proteins were reconstituted in Laemmli buffer, separated by a 1D-SDS-PAGE and treated as described above but without spiking-in the UPS2.

LC-MS/MS analyses were performed with a nano-HPLC system (nanoAcquity, Waters, Milford, MA, USA) online coupled to a LTQ Orbitrap XL ETD mass spectrometer (Thermo Fisher Scientific, San Jose, CA, USA). Peptide separation was achieved by RP-LC using a 90 min non-linear gradient (2–40% acetonitrile containing 0.1% formic acid). After MS full scan (positive mode, *m/z* 350 to 1,600, R = 60,000) MS/MS acquisitions of the six most abundant ions were automatically triggered when exceeding an intensity threshold of 3,000 counts. A dynamic exclusion was set for 120 sec limiting the list to a maximum of 500 entries. Fragmentation was conducted with the linear ion trap by collision induced fragmentation (CID, isolation window of 4 amu, normalized collision energy 35, activation time 30 ms, activation Q 0.25).

### Protein Identification and Quantification by Mass Spectrometry

The obtained MS raw data were processed using MaxQuant (Version 1.5.2.8) [22]. With the integrated search engine Andromeda [23] a target-decoy database search was performed. A reference set containing all annotated rat proteins (Uniprot, version 18th June 2015, 27,764 entries) in forward and reverse direction was used. Trypsin was set as endoprotease allowing for a maximum two missed cleavages. Acetylation of the protein N-terminus and oxidation of methionine were set as variable modifications whereas carbamidomethylation of cysteine was set as fixed modification. A precursor mass tolerance of 25 ppm was defined for first search. At least two peptides including one unique peptide were set as requirement for protein identification. The false discovery rate (FDR) for peptide and protein identification was controlled to be below 0.05. For protein quantification the MaxQuant integrated MaxLFQ algorithm for label-free protein quantification was used [24]. If not stated otherwise MaxQuant default values were used.

### Metabolomic Profiling and Quantification by Mass Spectrometry

Reconstituted microdialysate samples from 12–24 h after injury (n = 3 for BD, n = 6 for STW, 50 μL each) were precipitated by adding 500 μL of ACN. The samples were centrifuged, evaporated and reconstituted as described by Hoeke *et al*. [25].

LC-MS analysis was conducted on a Thermo Fischer Orbitrap Velos system using ESI positive ionization and UHPLC separation as described previously [25]. For details see Supporting Information. In addition, full scan acquisition (*m/z* 100–800, resolution = 30,000) using identical MS parameters was used for quantitative analysis. Data analysis was performed by XCMS online [26] using “HPLC / Orb (136)” parameters. Detailed information on the XCMS settings were described in S1 Text.

Data obtained by XCMS analysis was confirmed by targeted LC-MS analysis using AbsoluteIDQ p150 kit (BIOCRATES Life Sciences AG, Innsbruck, Austria) to quantify 163 metabolites as described elsewhere [21]. All experiments were carried out on an Agilent 1100 series binary HPLC system (Agilent Technologies, Waldbronn, Germany) coupled with an 4000 QTRAP^™^ linear ion trap mass spectrometer (AB Sciex, Concord, Canada) equipped with a TurboIon spray source. The FIA-analysis was performed with MS running solvent at ambient temperature. Quantification was achieved by multiple reaction monitoring (MRM) detection in combination with the use of stable isotope-labeled and other internal standards. Data evaluation for quantification of metabolite concentrations was performed with the MetIQ^™^ software package.

### Bioinformatics

Cell number was given as mean ± standard deviation (SD) and statistical significance was tested by Student t-test. The level of significance was set at p < 0.05.

ELISA data were statistically analyzed with the Kruskal-Wallis test. The Mann-Whitney test was used to detect post hoc statistical significance between selected groups. Statistical significance was set at p < 0.05.

All quantitative values for proteins gained from mass spectrometry were log10-transformed and median normalized, first between all corresponding fractions of each sample and afterwards between all samples. Proteins with quantitative values in at least three replicates of one condition and in none of the other were considered to be unique for the corresponding condition. Proteins with quantitative values in at least three replicates of both conditions were considered as quantified. To be treated as significantly higher abundant the log10-ratio in comparison of both conditions must be > 0.3 with a p-value of less than 0.05 (two-tailed, unpaired student’s t-test).

Protein clustering was based on Gene ontology (GO) assignments (GO:BP_FAT, GO:CC_FAT and GO:MF_FAT) using DAVID Bioinformatics Resources 6.7 [27] and refined by manual literature research. For an enrichment analysis a list of known extracellular rat proteins (evidences gained by mass spectrometry) provided by the ExoCarta database [28] (release date 29th July 2015) was used as background list. A principle component analysis (PCA) was conducted by using R and the package ggbiplot [29, 30]. Additionally, the quantitative protein data were analyzed through the use of QIAGEN’s Ingenuity^®^ Pathway Analysis (IPA^®^, QIAGEN Redwood City, www.qiagen.com/ingenuity). The log10-ratios and p-values of all quantified proteins were included. Unique proteins were assigned to a log10-ratio of 1 or -1, respectively, and p = 0.01.

## Results and Discussion

Bone repair is a highly complex process requiring the synchronization of multiple biological pathways to restore tissue integrity. Activation of these pathways gives rise to unique wound and fracture microenvironment that consists of cells and ECM including a complex milieu of enzymes, growth factors, and cytokines that control the tissue repair response. Thus, the real therapeutic challenge in enhancing bone healing is to provide an ideal microenvironment for optimal cell migration and proliferation. Nevertheless, the whole process in its complexity is still poorly understood. Even though the significance of single proteins is determined, for a thorough understanding of the whole process it is relevant to identify and quantify the involved molecules at their site of action. It is well known that the initial inflammatory stage is essential for later consolidation [31–33]. Hence, the aim of this study was the identification and quantification of chemokines, cytokines and further proteins involved in the early stage of bone healing as well as metabolites that could provide further insight into the metabolic adaptions following a fracture.

Because the concentration of the cytokines in plasma does not represent the concentration in the injured tissue, quantification at the wound site is highly desirable [34, 35]. Microdialysis is an ideal sampling technique as it provides the capability to monitor these proteins in real time in their surrounding milieu. However, chemokines and cytokines are small molecules present in low concentration. In addition, the BSA added to the perfusate to increase the recovery of proteins cover the signals from the chemokines and cytokines. Taken together, this leads to an underrepresented identification and quantification of those proteins in a nanoLC-MS/MS approach [21]. Hence, ELISAs were used to quantify the cytokines in the dialysate because of their acceptable specificity, sensitivity, and the ease of performance.

Even though microdialysis is a minimally invasive technique, there is also evidence that the insertion of the catheter into the tissue can change the interstitial concentration of cytokines [36]. But since BD as well as STW are dramatic injuries the effect of implantation of the catheter in the present experiment is expected to be negligible compared to the inflammation and thus the cytokine response caused by the injuries.

### Clinical Findings

Forty three rats survived the surgery without any complications. One animal died due to severe anemia during the surgery. Three animals died during the experiment without any identifiable reason. The dialysates of eleven rats with BD and those of 9 rats with STW each sampled at 0–12 h and 12–24 h could be used for ELISA analysis.

### Determination of Cytokines and Growth Factors with ELISA

In total the abundance of 15 cytokines and growth factors were investigated in the dialysate from BD and STW. Under the experimental conditions six cytokines and growth factors were not detectable (BMP-2, VEGF, PDGF-BB, IL-1α, IL-1β, and TNF-α) which might be due to several reasons. First, the recovery of proteins depends reciprocally on their molecular weight [37]. Although we used a cut-off of 100 kDa the larger cytokines VEGF (MW appr. 40 kDa) or PDGF-BB (MW appr. 30 kDa) seem to be unable to cross the membrane with sufficient recovery which is in line with the observation that an acceptable recovery will only be attained if the molecular weight of the proteins is lower than approximately one-fourth of the membrane cut-off [38]. However, we used this cut-off to avoid the recovery of serum proteins and to avoid an influx of perfusion fluid into the wound area which we have seen using catheter with larger pore size. Second, the recovery is also controlled by the concentration of the protein. A higher concentration of the protein means a higher concentration gradient driving the transfer over the membrane [39]. Third, since it is well known that most cytokines are usually found in low picomolar concentrations [14] we cannot exclude that the concentration of these investigated proteins in the wound fluid was already below the detection limit. Forth, the composition of the perfusion fluid, the flow rate and the area of the membrane are further criteria influencing the recovery of proteins [40]. The optimization of the perfusion fluid composition and the flow rate we described previously [13]. But we cannot exclude that other flow rate or buffer composition could result in detectable protein concentrations. The length of the used membrane was 4 mm, because the diameter of the rat femur is about 3–4 mm. The sampling of proteins from BD could thus be limited to the area of the BD because the catheter tip does not stick out into the soft tissue. Despite these challenges and limitations, microdialysis can be used to receive both time-dependent qualitative and quantitative data from the site of injury which cannot be achieved by other discontinuous methods.

**Fig 1:**
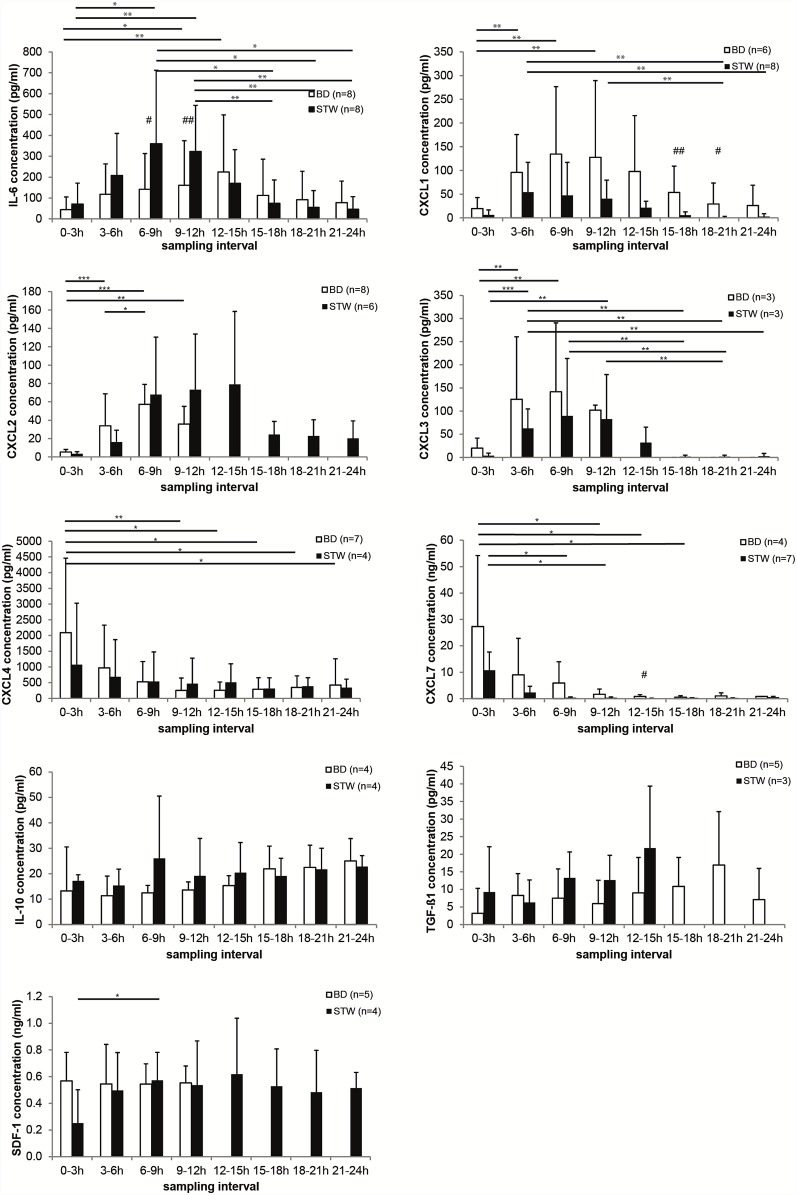
Concentration of chemokines and cytokines in the dialysate from bone defect (BD) and soft tissue wound (STW) 0–24 h after injury. Samples were analyzed by ELISA. Values are mean ± SD. IL-6, interleukin-6; CXCL2, chemokine (C-X-C motif) ligand 2; CXCL7, chemokine (C-X-C motif) ligand 7; CXCL3, chemokine (C-X-C motif) ligand 3; CXCL1, chemokine (C-X-C motif) ligand 1; CXCL4, chemokine (C-X-C motif) ligand 4; IL-10, inteleukin-10; TGF-β1, transforming growth factor-β1; SDF-1, stromal-cell derived factor-1. * p < 0.05, ** p < 0.01, *** p < 0.001.

For nine markers assessed, concentration profiles could be obtained (Fig 1). IL-6, CXCL1, CXCL2, and CXCL3 showed an increase in concentration after surgery and a re-equilibration to baseline after 24 h. The concentrations of CXCL4 and CXCL7 decreased immediately after surgery, suggesting a release of the proteins from activated cells. SDF-1, TGF-β1, and IL-10 did not show any changes in concentration over the first 24 h after surgery.

The key function of IL-6 is mediation of the acute phase response. After release at the site of injury IL-6 moves to the liver through the bloodstream and induces the synthesis of acute phase proteins such as C-reactive protein, fibrinogen, serum amyloid A, haptoglobin, and α1-antichymotrypsin (SerpinA3) in hepatocytes [41]. In the present series, IL-6 concentration in the dialysate peaked earlier in the STW than in the BD. The highest concentration of IL-6 in BD was detected 12–15 h after surgery. In contrast, for STW the maximum was reached at 6–9 h. The early increase after the injury underlines the role of IL-6 in the acute phase response. With the Kruskal-Wallis test the differences in the time response of the IL-6 concentrations in STW was statistically significant different (p = 0.006). In the intervals 6–9 h and 9–12 h the absolute IL-6 concentration was significantly higher in STW than in BD (6–9 h: p = 0.047; 9–12 h: p = 0.007). A similar time response was found in UVB-irradiated skin. IL-6 increased rapidly after 8–16 h and declined to baseline after 24 h [10].

CXCL1, CXCL2, and CXCL3 are all potent chemoattractants for neutrophils and are considered to play key role in neutrophil infiltration in inflammatory diseases [42]. All three CXCLs have a similar ability to induce chemotaxis for neutrophils *in vitro* and *in vivo* [43, 44]. However, the contribution of each CXCL to neutrophil infiltration in vivo seems to depend on the amount of each CXCL at the site of inflammation [45]. In addition, in rat the proportion of each CXCL at the site of inflammation depends on the type of inflammation [42].

CXCL1 expression has been demonstrated for platelets but also endothelial cells, neutrophils, monocytes, and macrophages [46–48]. In BD the highest CXCL1 concentration was detected 6–9 h after surgery. In STW the highest CXCL1 concentration was seen 3–6 h after surgery. For both defect scenarios the time response showed significant differences (BD: p = 0.007; STW: p = 0.004) resulting in p-values p < 0.01 determined by a post hoc test. In the intervals 15–18 h and 18–21 h the differences between BD and STW were statistically significant (15–18 h: p = 0.005; 18–21 h: p = 0.025). CXCL1 is constitutively expressed by endothelial cells, megakaryocytes in the bone marrow, and to a lesser extent by osteoblasts [49, 50]. This might explain the higher CXCL1 concentration in BD compared to STW where the bone and thus the bone marrow were not injured.

The macrophage inflammatory protein 2 alpha (CXCL2) is not stored in platelets but expressed, among other cells, by macrophages and monocytes [51]. In contrast to the IL-6 concentration, CXCL2 in the dialysate peaked earlier in BD than in STW. The highest CXCL2 concentration in BD was detected 6–9 h after surgery while the maximum level in STW was detected 12–15 h after surgery. However, 12 h after surgery no CXCL2 was detectable in BD. The post hoc test revealed significant differences between 0–3 h and 3–6 h (p < 0.001), 6–9 h (p < 0.001) and 9–12 h (p = 0.002). The total CXCL2 concentration was higher in STW than in BD.

The CXCL3 concentration was highest 6–9 h after surgery in BD as well as in STW. In BD CXCL3 was not detectable anymore after 12 h. Nevertheless, the time response was statistically significant different in BD (p = 0.013) and STW (p < 0.001) and resulted in p-values from post hoc tests below p < 0.01.

For all three CXCL types we found a similar time response. The concentration increased immediately after injury, peaked early and then decreased after 12–15 h. For CXCL1 and CXCL2 the inflammatory response was comparable to that in a rat burn model. In the latter, serum CXCL1 increased rapidly after 3 h and then decreased slowly while CXCL2 showed a rapid increase immediately after the injury with a peak at 6 h and then a dramatic decrease between 12 h and 24 h [52] similar to the missing CXCL2 in BD after 12 h in the current experiment.

Platelet factor 4 (CXCL4) and pro-platelet basic protein (CXCL7) are abundant chemokines in the α-granules of platelets that are released during platelet activation [53]. This is in accordance with the concentration profile determined for CXCL4 and CXCL7 in the present study. In BD and STW the CXCL4 concentration decreased after surgery and reached steady-state level 9–12 h after surgery. The difference in time response was not statistically significant different (BD: p = 0.099; STW: p = 0.981).

For CXCL7 the concentration decreased from 27.3 ng/mL (SD 26.8 ng/mL) at 0–3 h to 0.8 ng/mL (SD 0.68 ng/mL) at 12–15 h and remained at low level for the rest of the sampling intervals in BD. In STW the steady state level was reached with the 6–9 h interval. However, the time response for STW is statistically significant different (p = 0.02).

A third time-dependent concentration profile was found for IL-10, TGF-ß1 and SDF-1. For these cytokines, the concentration in the samples did not change over time. IL-10 is an anti-inflammatory cytokine and thus it will be upregulated at the end of the inflammatory stage. On the other hand, there is evidence that the recovery of IL-10 is low because of a larger hydrodynamic radius compared to other cytokines [40, 54].

TGF-β1 is important in all wound healing stages. In the inflammatory stage it is released by platelets and facilitates the recruitment of additional inflammatory cells and MSCs [3, 55]. After that TGF-β1 is being expressed by other cells over 21 days during fracture healing [3]. In STW TGF-β1 was only detectable until 12–15 h after surgery. Until then the concentration slightly increased from 9.2 pg/mL (SD 12.9 pg/mL) to 21.7 pg/mL (SD 17.6 pg/mL). For BD the TGF-β1 concentration ranged from 3 to 17 pg/mL over 24 h.

The concentration of SDF-1 in the BD samples remained constant over the whole 24 h. In STW samples the SDF-1 concentration increased after the first interval and post hoc test identified a statistically significant increase from 0–3 h to 6–9 h (p = 0.017). SDF-1 recruits MSCs during endochondral bone repair. An increase of the SDF-1 mRNA was not observed before day 2 in a mouse segmental bone graft model [56]. Thus, the constant local protein level over the first 24 h after creation of a bone defect may indicate an early steady state before systemic upregulation.

The ELISA results show that microdialysis is a suitable method to determine the concentration profiles of various proteins associated with the inflammatory stage of the healing process. In addition, we could show differences between BD and STW. This is an important fact because it is now possible to identify dysregulated proteins in delayed wound and fracture healing. Second, influencing the expression or the release of these proteins could result in an improved healing process.

**Fig 2:**
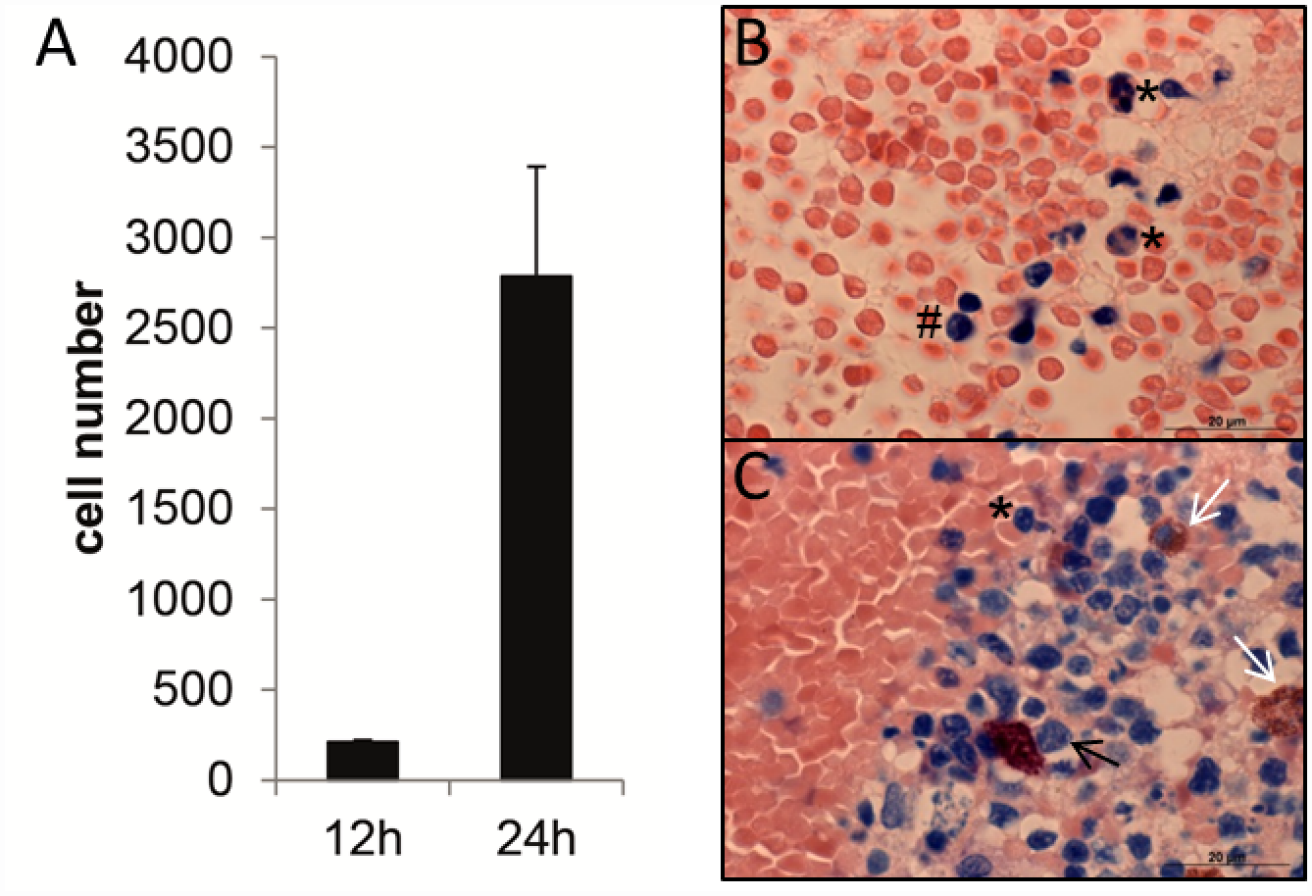
Analysis of the fracture hematoma from BD 12 h and 24 h after surgery. (A) Number of mononucleated and multinucleated cells in the fracture hematoma. Cells were counted in 10 randomly distributed squares per section on three subsequent slices per fracture hematoma. (B) Pappenheim staining of fracture hematoma 12 hours after surgery. (C) Pappenheim staining of a fracture hematoma 24 h after surgery. Magnification 100x. *, neutrophil granulocyte; #, monocyte; black arrow, basophil granulocyte; white arrow, eosinophil granulocyte.

The number of polymorphonuclear cells in the fracture hematoma from BD was significantly lower after 12 h compared to 24 h indicating the beginning of the inflammatory stage (Fig 2A). After 12 h we could mostly detect neutrophil granulocytes and some monocytes. In the fracture hematoma 24 h after surgery increased number of neutrophil granulocytes but also eosinophil and basophile granulocytes were seen (Fig 2B and 2C). The increase in polymorphonuclear cells corresponds to the time line of cellular migration during healing. After coagulation neutrophils are the first cells arriving at the wound site peaking after 24–48 h. They are responsible for phagocytosis and wound debridement and thus they decrease the rate of infections [57, 58]. Neutrophils are important cells because they also produce and express chemokines which chemoattract further immune cells and as a result the healing process is going on. We could only analyze the fracture hematoma from BD because no hematoma was formed in a STW.

### Wound Fluid Proteomics

The combination of microdialysis with proteomics provides additional information about the composition of the fracture hematoma and could be used in order to identify potential biomarkers of bone healing. In the present experiment, the total amount of protein recovered from BD or STW by microdialysis was too low for HPLC-MS/MS analysis. We identified only 28 proteins, most of them ubiquitously produced in cells (S1 Table). We therefore used the proteins adsorbed to the tip of the microdialysis catheter membrane for proteomic analysis. This allows for effective and reproducible sampling as has been shown recently [21]. By using this approach an overall number of 513 proteins could be identified in a comparative analysis of both defect scenarios. Among those 273 proteins could be reliably quantified (S2 Table).

**Fig 3:**
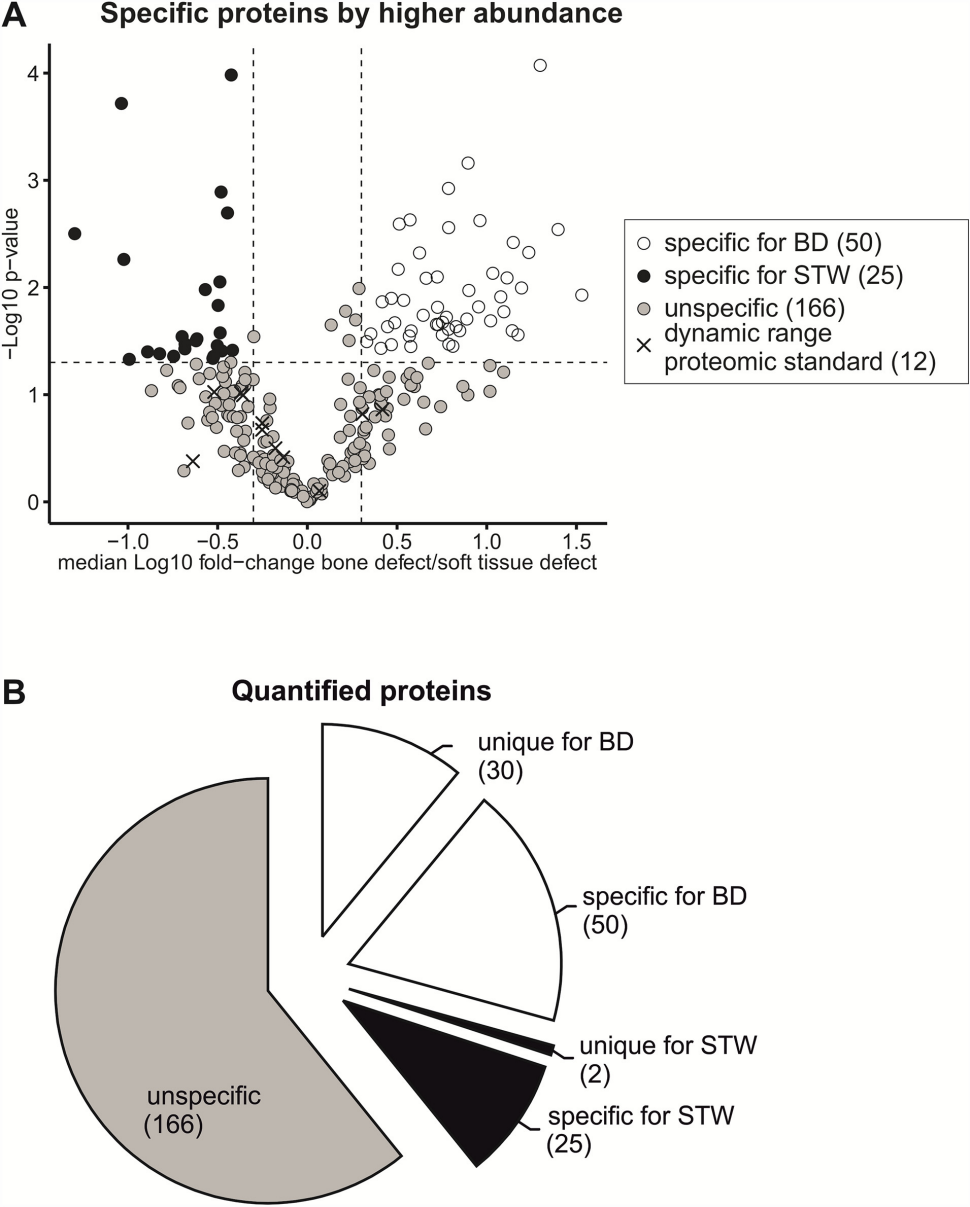
Comparative analysis of protein adsorbates sampled from bone defect (BD) and soft tissue wound (STW) at 12 h after injury. n = 4 for each defect scenario. (A) Volcano plot of proteins quantified in both defect scenarios: Data points represent the Log10-transformed ratios of the mean quantitative values gained from BD or STW and the -Log10-transformed p-value of the comparative analysis. (B) Proportions of unique and specific proteins for a certain wound scenario among all quantified proteins. The numbers in brackets represent the number of proteins assigned to the corresponding groups.

To identify significant alterations in the protein composition of fluids from different wound scenarios, two different approaches were pursued: (i) identification of unique proteins for a certain wound scenario and (ii) a determination of significant higher abundance of proteins for a certain wound scenario. To be considered as *unique*, a protein must be quantified in at least three of four replicates of one wound scenario but in none of the other. To be considered as *significantly higher abundant*, the Log_10_-ratio of the protein abundance in both defect scenarios must exceed 0.3 (= 1-fold increase) with a p-value of p < 0.05 (student’s t-test). The 19 quantified proteins of the spiked-in Proteomics Dynamic Range Standard served as control (Fig 3A). None of the spiked-in standard proteins was identified as significantly regulated, indicating a successful quantification and normalization procedure. By applying both described strategies to all 273 reliably quantified proteins, 107 of them could be assigned to a certain wound scenario (Fig 3B). The number of specific proteins for the bone defect group (BD, 30 unique, 50 by higher abundance) was notably higher than the number for the soft tissue wound group (STW, 2 unique, 25 by higher abundance). This difference appears plausible because the BD scenario has all features of the STW but with an additional fracture of the femur.

**Fig 4:**
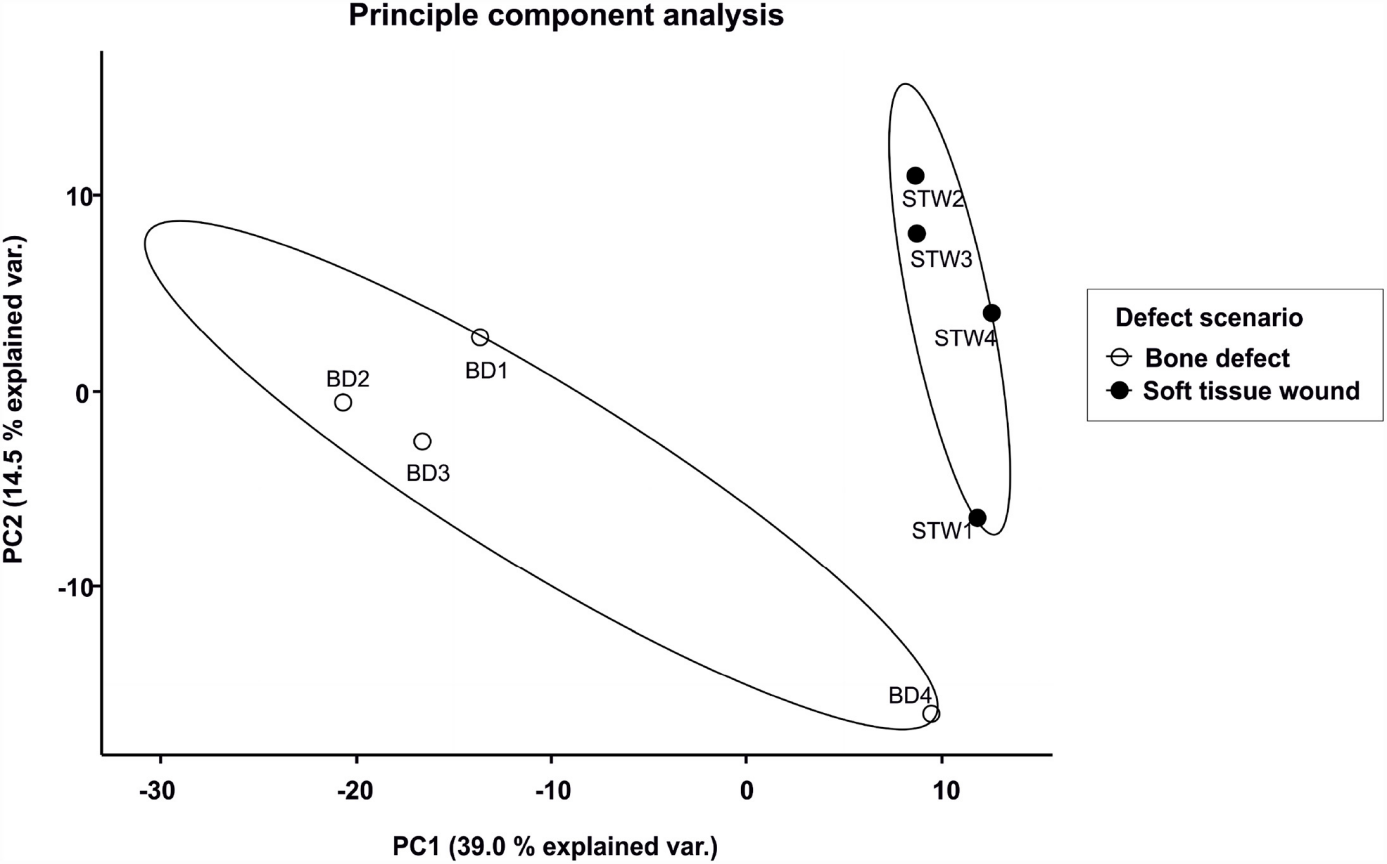
Principle component analysis (PCA). n = 4 for each defect scenario. PCA based on protein quantitative values gained from each replicate of bone defect (BD) and soft tissue wound (STW) at 12 h after injury.

To confirm a specific protein profile of the sampled wound fluids for a given defect scenario a principle component analysis (PCA) of all quantified proteins was conducted (Fig 4). The two defect groups could be separated by this analysis.

In summary, by (i) identifying unique proteins for both defect scenarios, (ii) showing a significant higher abundance of several proteins depending on certain wound scenarios and (iii) by the clear separation of both defect types in the PCA, defect-specific protein profiles can be considered.

### Identification of Early Wound or Fracture Healing Markers

**Fig 5:**
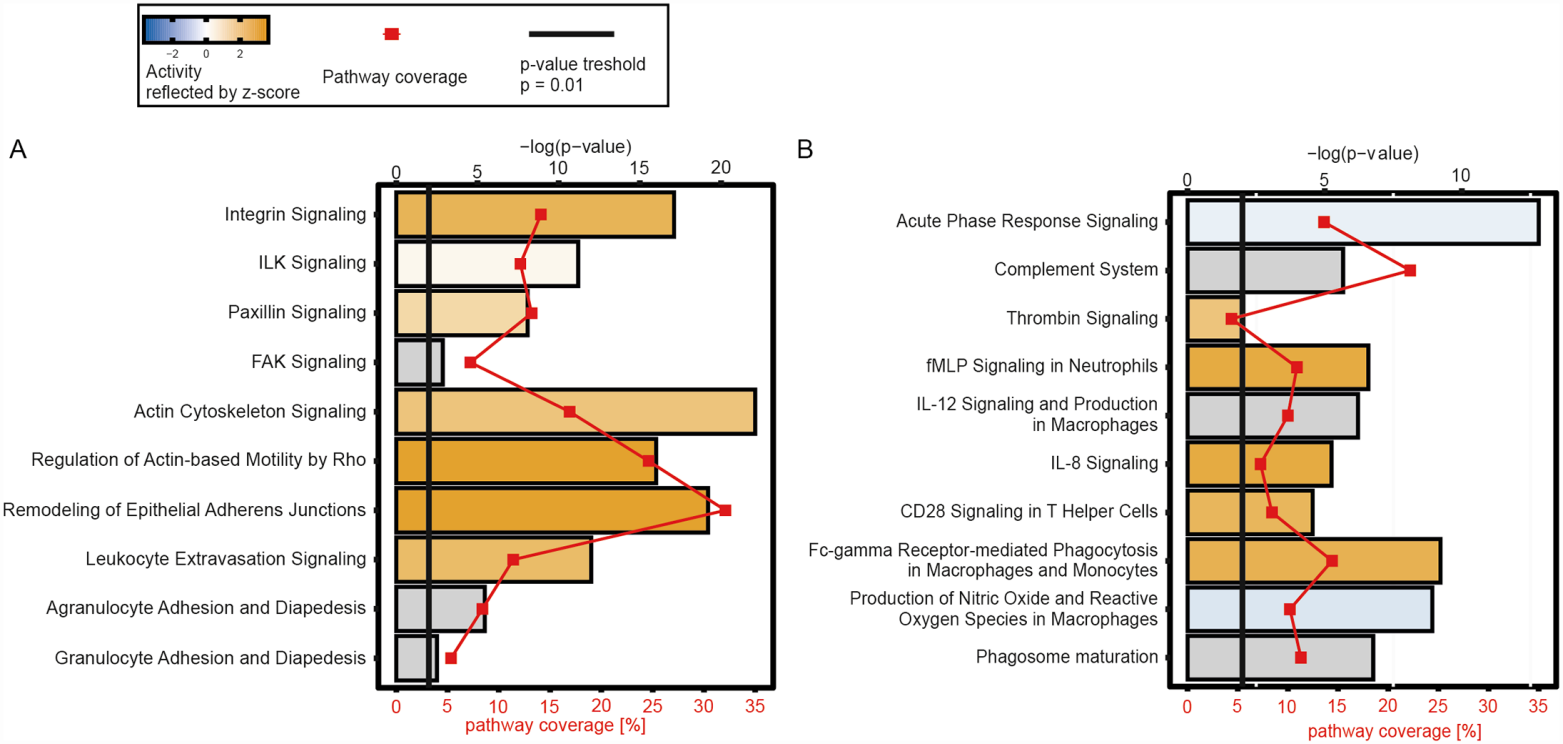
Signaling pathway analysis. n = 4 for each defect scenario. Regulation of selected pathways focusing on cellular migration (A) and immune response (B) revealed by Ingenuity Pathway Analysis (IPA). The quantitative values of all proteins were used. Z-score reflects the pathway activity with orange indicating an increased activity in the bone defect group and blue a decreased activity, respectively.

To gain deeper insight into the biological potential of the wound fluids all quantified proteins were assigned to functional clusters based on GO-term assignment and manual literature research. An enrichment analysis of all quantified proteins revealed, among others, an increased presence of proteins being involved in proteinase inhibition (enrichment score = 3.8), wounding and inflammatory response (3.29), apoptosis (2.79), tissue regeneration (2.46), blood coagulation (2.43) and lymphocyte chemotaxis and activation (2.06) (S3 Table). Moreover, a signaling pathway analysis revealed several enriched and activity-altered processes (S4 Table). A selection of higher hierarchical signaling pathways with a focus on cellular adhesion and migration (Fig 5A) and immune response (Fig 5B) is shown. Several pathways are predicted to be in a higher activity state in the bone defect group. This could indicate an increased invasion of immune cells to the wound area after a femur fracture compared to the soft tissue wound. The acute phase response signaling is one of only two pathways with a decreased activity. This confirms the finding of an earlier IL-6 response in the soft tissue wound.

Since an impaired wound healing is believed to be based on an improper inflammatory response, we manually clustered all quantified proteins to early wound healing mechanisms including ECM proteins, immune modulators, proteases and protease inhibitors as described before [19] (S5 Table). Tables 2–5 lists the top 15 most abundant proteins including all unique or specific proteins for a certain wound scenario, assigned to those clusters.

**Table 2:** Identified proteins clustered to extracellular matrix proteins. Identified proteins from proteomic analysis at 12 h after injury. Gene symbols and protein names correspond to Uniprot Knowledgebase. Quantitative values are given as Log10-transformed fold changes BD/STW. Significances are indicated as asterisks with * p-value < 0.05, ** p-value < 0.01 and *** p-value < 0.001. n = 4 for each defect scenario.

Gene symbole	Protein name	Log_10_ BD/STW
OLFM4	Olfactomedin 4	***unique for BD***
LGALS3	Galectin-3	***unique for BD***
ANXA2	Annexin A2	**0.79** *
FN1	Fibronectin	**-0.42** ***
VTN	Vitronectin	-0.44
FGA	Fibrinogen alpha chain	**-0.49** **
FGB	Fibrinogen beta chain	-0.38
FGG	Fibrinogen gamma chain	**-0.49** *
AHSG	Alpha-2-HS-glycoprotein	**-0.50** *

**Table 3:** Identified proteins clustered to immune modulators. Identified proteins from proteomic analysis at 12 h after injury. Gene symbols and protein names correspond to Uniprot Knowledgebase. Quantitative values are given as Log10-transformed fold changes BD/STW. Significances are indicated as asterisks with * p-value < 0.05, ** p-value < 0.01 and *** p-value < 0.001. n = 4 for each defect scenario.

Gene symbole	Protein name	Log_10_ BD/STW
NCF2	Neutrophil cytosol factor 2	***unique for BD***
NCF4	Neutrophil cytosol factor 4	***unique for BD***
CYBB	Cytochrome b-245, beta polypeptide	***unique for BD***
LCP1	Lymphocyte cytosolic protein 1	**1.53** *
ITGB2	Integrin beta	**1.17** *
CORO1A	Coronin-1A	**0.96** **
YWHAZ	14-3-3 protein zeta/delta	**0.65** *
ANXA1	Annexin A1	0.49 *
NGP	Neutrophilic granule protein	**0.42** *
S100A9	Protein S100-A9	**0.33** *
S100A8	Protein S100-A8	0.32
CRP	C-reactive protein	-0.22
C3	Complement C3	-0.30
CFH	Complement factor H-related protein	**-0.68** *
PRDX2	Peroxiredoxin-2	**-0.42** *

**Table 4:** Identified proteins clustered to proteases. Identified proteins from proteomic analysis at 12 h after injury. Gene symbols and protein names correspond to Uniprot Knowledgebase. Quantitative values are given as Log10-transformed fold changes BD/STW. Significances are indicated as asterisks with * p-value < 0.05, ** p-value < 0.01 and *** p-value < 0.001. n = 4 for each defect scenario.

Gene symbole	Protein name	Log_10_ BD/STW
PSMB10	Proteasome subunit beta type-10	***unique for BD***
PR3	Proteinase 3	***unique for BD***
MMP8	Matrix metalloproteinase 8	***unique for BD***
MMP9	Matrix metalloproteinase 9	0.46
NAPSA	Napsin	**1.03** **
Cat-G	Cathepsin G	**0.73** *
EL	Neutrophil elastase 2	0.31
F2	Prothrombin	-0.11
PLG	Plasminogen	**-0.50** *

**Table 5:** Identified proteins clustered to protease inhibitors. Identified proteins from proteomic analysis at 12 h after injury. Gene symbols and protein names correspond to Uniprot Knowledgebase. Quantitative values are given as Log10-transformed fold changes BD/STW. Significances are indicated as asterisks with * p-value < 0.05, ** p-value < 0.01 and *** p-value < 0.001. n = 4 for each defect scenario.

Gene symbole	Protein name	Log_10_ BD/STW
AGT	Angiotensinogen	0.26
SERPINB1A	Serine protease inhibitor EIA	**1.10** *
SERPINA1	Alpha-1-antiproteinase	-0.35
SERPINA3K	Serine protease inhibitor A3K	-0.21
SERPINA3L	Serine protease inhibitor A3L	-0.19
SERPINA3N	Serine protease inhibitor A3N	-0.05
SERPINC1	Protein Serpinc1	-0.17
SERPIND1	Heparin cofactor 2	-0.37
ITIH2	Protein Itih2	-0.51
ITIH3	Inter-alpha-trypsin inhibitor heavy chain H3	-0.20
ITIH4	Inter alpha-trypsin inhibitor, heavy chain 4	-0.54
FETUB	Fetuin-B	-0.39
A1I3	Alpha-1-inhibitor 3	**-0.48** *
MUG1	Murinoglobulin-1	**-0.52** *
AMBP	Protein AMBP	**-0.75** *

### Different Abundance of ECM Proteins in Wound Fluids

Proteins forming insoluble fibers are essential for primary wound closure. Moreover, they provide a provisional ECM for the migration and adhesion of immune cells and re-epithelization. Overall, nine proteins could be assigned to the ECM protein cluster (Table 2). With fibrinogen alpha and gamma chain (FGA, FGG) and fibronectin (FN1) two were specific by higher abundance for the STW group. The mature fibrin and FN1 were shown to be involved in forming a provisional ECM [59, 60]. The earlier formation of a provisional ECM is in line with the finding of an earlier response of the acute phase mediator IL-6 in the STW group compared to the BD group. However, alpha 2 HS-glycoprotein (AHSG) was also identified specific by higher abundance for the STW group. This protein was shown to be negatively correlated to the acute wound healing phase [61]. Galectin-3 (LGALS3), olfactomedin 4 (OLFM4), and annexin A2 (ANXA2) were specific for BD. LGALS3 has been shown to be involved in the acute inflammatory response including chemoattraction of monocytes and macrophages, as well as neutrophil activation and adhesion [62, 63]. OLFM4 has been found in granules of neutrophils [64, 65] and appears to be an important regulator of the innate immunity against bacterial infections [66]. On the other hand, OLFM4 was reported in higher abundance in chronic non-healing wounds [19]. ANXA2 is expressed in endothelial cells, macrophages and mononuclear cells [67]. Recent studies suggest that secreted ANXA2 can activate macrophages to secret IL-1, IL-6, and TNF-α, and can recruit and activate immune cells to the site of infection [68, 69].

### Different Abundance of Immune Modulators in Wound Fluids

The balance of the immune response has a critical role in influencing the outcome of wound and fracture healing. Both, an inadequate primary defense against pathogens invading the wound and an unregulated activation of the immune system overwhelming the protective capacity of the tissue could lead to a non-healing wound or fracture nonunion. 27 proteins with immunomodulatory capabilities were quantified in the wound fluids underlining the crucial role of a proper immune response at the wound or fracture site. Ten of these proteins were either unique or significantly higher abundant in the BD group (Table 3). This includes cytosolic proteins of neutrophils (neutrophilic granule protein, NGP; neutrophil cytosol factor 2, NCF2; and neutrophil cytosol factor 4, NCF4) indicating a rapid migration of these cells into the wound area. This is supported by a higher abundance of integrin beta (ITGB2) and protein S100-A9 (S100-A9). Both proteins are involved in neutrophil migration towards wound sites [70, 71]. Neutrophils are known to be a key player in the early innate immune response [72]. Additionally, with the lymphocyte cytosolic protein 1 (LCP1) and coronin-1A (CORO1A) two proteins crucial for T cell activation and migration were found to be specific for the BD [73, 74]. Moreover, annexin A1 (ANXA1) was upregulated in the BD group. This protein is involved in several pathways of the immune response but also has a crucial role for resolving the inflammatory response in later healing stages [19, 75]. For the STW group only two proteins were found to be upregulated in the immune modulating protein cluster. Of those, the complement factor H-related protein (CFH) was shown to be a downregulator of the alternative complement activation [76].

### Different Abundance of Proteases in Wound Fluids

One of the main functions of cells involved in the early immune response is the secretion of proteases either for defense, tissue remodeling or migration purposes. However, those proteases also lead to tissue damage, if the response is not resolved properly. Nine quantified proteins from the sampled wound fluids could be assigned to proteases (Table 4), including five of them being known to be secreted by neutrophils (cathepsin G, Cat-G; neutrophil elastase 2, EL; matrix metalloproteinase 8, MMP8; matrix metalloproteinase 9, MMP9; and proteinase 3, PR3) [77–79]. In inflammation the neutrophil proteases can activate or inactivate cytokines, activate specific receptors, and modify the activity of chemokines [80]. Five of nine proteins were unique or specific for the BD group, once more suggesting more intense immune response after a fracture as compared with a pure soft tissue wound. However, to the best of our knowledge, no influence on wound healing has yet been described for the BD-specific proteasome subunit beta type-10 (PSMB10) and napsin (NAPSA). Plasminogen (PLG) was the only protein identified in a higher abundance in the STW group. Besides its role in terminating blood coagulation it was shown to have a role in wound healing by remodeling of the ECM [81].

### Different Abundance of Protease Inhibitors in Wound Fluids

A balanced activity of immune-related proteases is, among other mechanisms, achieved by the secretion of protease inhibitors. 20 proteins assigned to this cluster were quantified in the wound fluids, showing the importance of proteinase inhibitors in the wound area (Table 5). This includes nine proteins of the serine protease inhibitor protein superfamily (serpin) and four Inter-alpha-trypsin inhibitor heavy chains. Despite its structural similarities serpins inhibit a variety of serine proteases being involved in manifold biological processes, including tissue remodeling [19, 82]. Inter-alpha-trypsin inhibitors are hyaluronic acid-binding proteins. By this binding they stabilize the ECM and prevent it from degradation [83]. Four wound scenario-specific proteins could be identified within this cluster. Serpin B1A –an inhibitor of the neutrophil-released Cat-G, EL and PR3 [84]–was the only protein with a higher abundance in the BD group. Alpha-1-inhibitor 3 (A1I3), its protein homologue murinoglobulin-1 (MUG1), and AMBP were specific for the STW group. A significant drop of both the mRNA expression in liver cells and the serum protein concentration of A1I3 could be shown within the first 24 h of the acute phase [85, 86].

### Different Abundance of Metabolites Elucidated by Metabolic Screening and Validated by Targeted Quantification

**Fig 6:**
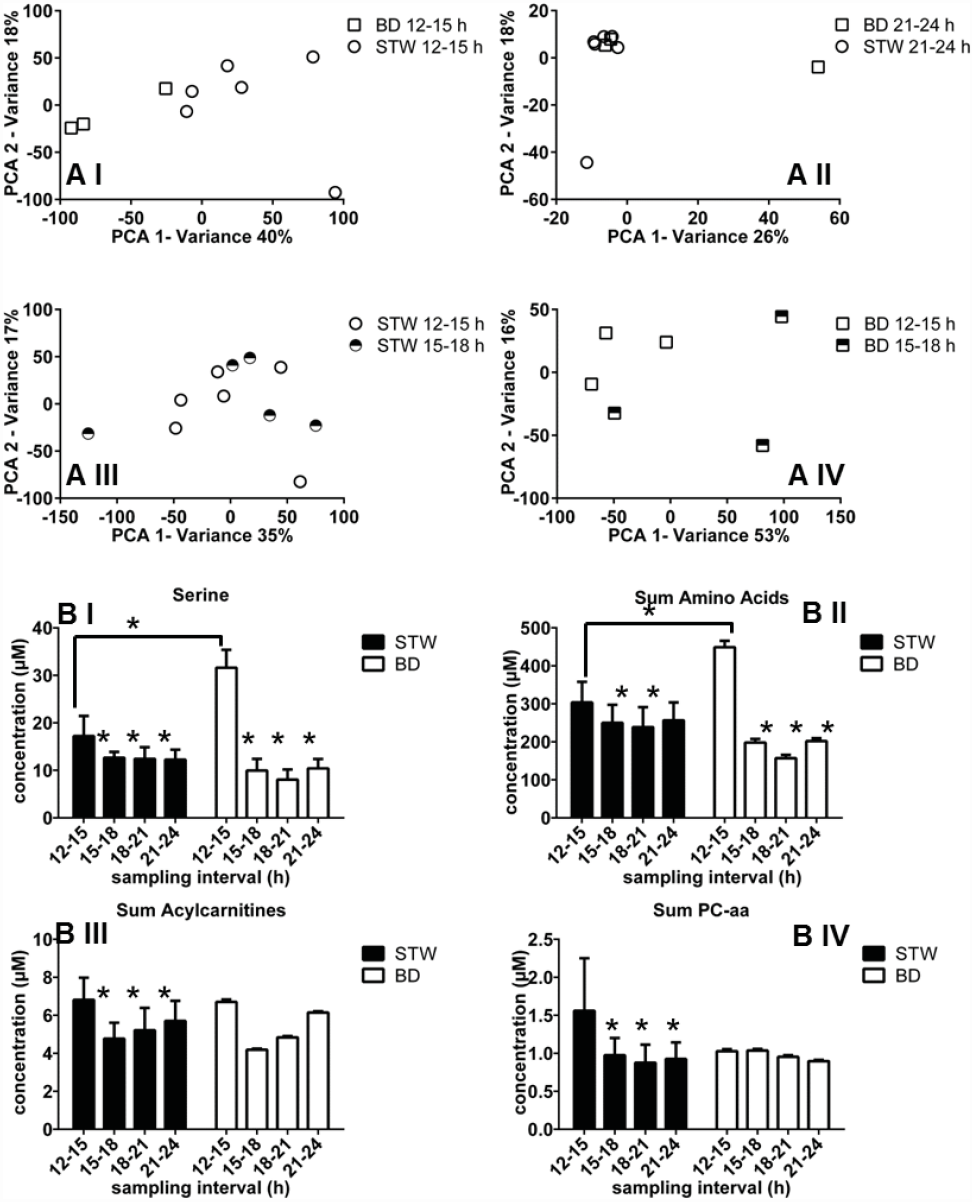
Metabolomic results in the dialysate from bone defect (BD) and soft tissue wound (STW) 12–24 h after injury. (A) PCA comparisons of BD vs. STW after 12-15h (I) and 21-24h (II) as well as monitoring of STW (III) and BD after 12-15h vs. 15-18h (IV). n = 6 for soft tissue wound and n = 3 for bone defect. (B) Absolute concentration of serine in STW and BD wound fluids (I). Summarized concentrations of all monitored amino acids, acylcarnitines, and phosphatidylcholines-aa are shown in II-IV. Bar charts are depicted as mean + SD, * indicates a p-value < 0.05. n = 6 for soft tissue wound and n = 2 for bone defect.

Metabolic screening and subsequent targeted validation revealed significantly different regulations of metabolite concentrations between BD and STW, as well as time-dependent differences. The largest differences between both wound fluids could be obtained by metabolic screening of the sampling interval 12–15 h (Fig 6AI–6AIV). This could also be verified by targeted approach indicated by highest amount of significantly regulated metabolites. Among other mechanisms, metabolic screening revealed significant time-dependent alterations of several amino acid pathways such as serine, tryptophan, histidine, arginine, and proline metabolism in BD and STW fluids. Targeted quantification of these potential key metabolites for wound fluid differentiation confirmed these findings with significant differences. In general most of the monitored amino acids in BD wound fluid showed a significant higher concentration at early time-point compared to STW wound fluid (Fig 6BI and 6BII). Since the investigated BD is the more severe type of inflammation this is in accordance to Yang *et al*. reporting elevated levels of e.g. serine in wound dialysates in early phase of inflammation as a function of immune response. After 24h elevated amino acid levels decreased to control level confirming our present data [87].

Another finding in metabolic screening was a variation of the carnitine shuttle. Quantification of 41 acylcarnitines in examined wound fluids revealed significant time-dependent differences between BD and STW samples (Fig 6BIII). Thereby it is noteworthy that concentrations of STW and BD wound fluids showed a similar appearance resulting in highest levels at 12–15 h followed by a significant decrease of carnitine concentrations and subsequent alignment to initial conditions. This course of acylcarnitine levels could be also explained by immune responses as also indicated by cytokine profiling (Fig 1) [88]. A differentiation of carnitine levels between STW and BD fluids was not obtained.

Furthermore, the regulation of glycerophospholipid metabolism of BD and STW fluids obtained by metabolic screening could be verified using a targeted quantification approach. Targeted MS analysis showed higher concentrations of glycerophospholipids in STW after 12–15 h compared to BD (Fig 6BIV). The deeper insight using targeted quantification elucidated a major influence of diacyl-bonded phosphatidylcholines (PC-aa) compared to acyl-alkyl (PC-ae) or lyso-phosphatidylcholines (lyso-PC) regarding the fluid-relevant differences. Two major potential targets for a differentiation of STW- and BD-wound fluid are PC-aa 34 (monitored via PC-aa 34:1, 34:2, 34:3, and 34:4) and PC-aa 36 (monitored via PC-aa 36:0, 36:1, 36:2, 36:3, and 36:4, 36:5, and 36:6) two integral components of cell membranes which showed significant higher levels after 12–15 h in STW compared to BD wound fluid.

## Conclusion and Outlook: Potentials of Combined Sampling of Wound Fluids

In this study we established a combined sampling by a minimally invasive technique with screening of two basic wound scenarios (bone defect and soft tissue wound) within the first 24 h after the injury. We were able to clearly differentiate between both wound scenarios and identify BD-specific features at all three levels of analysis, i.e. bone specific cytokine responses and distinct protein and metabolite profiles. Moreover, this differentiation is based on the capability to quantify cytokines, proteins and metabolites that can serve as a hallmark for a physiologic or dysregulated wound or fracture healing. The response of the acute phase mediator IL-6 was delayed in the samples from BD compared to STW. On the other hand, when using proteomics of the wound fluid, the negative acute phase markers AHSG and A1I3 were more abundant in STW. The neutrophil chemoattractants CXCL1, CXCL2 and CXCL3 were quantified with either a higher or earlier response in the BD dialysates. This finding was supported by wound fluid proteomics which revealed a higher abundance of neutrophil cytosolic proteins (NGP, NCF2 and NCF4) as well as by the presence of neutrophil migration factors (ITGB2 and S1009A). Moreover, neutrophil-released proteases (Cat-G, MMP8 and PR3) were enriched in the wound fluids of the BD group. With regard to an identification of a variety of protease inhibitors in both wound scenarios but with only one specific inhibitor for the BD group, a more intense innate immune response can be assumed after the femur fracture compared to the STW control group. This is supported by a higher abundance of fibrin and fibronectin constituting of a provisional ECM composed of fibrin and fibronectin, which were higher abundant in the STW group compared to the BD. Recently, non-healing wounds are mainly thought to be linked to an imbalanced and persistent immune response that overwhelms the tissue protective capabilities [19, 72, 77]. Hence, the monitoring of immune modulators, proteases and their corresponding inhibitors could serve as markers for the wound and fracture healing progress.

Future studies employing combined sampling should focus on the comparative monitoring of healing and non-healing bone defects under different conditions that could reveal the molecular background of the healing progress. Moreover, this technique could be used for assessing the host response towards different bone graft or bone substitute materials. However, this sampling approach is not limited to immune-related questions since proteins involved in several biological processes, like sugar metabolism and cytoskeleton organization, could be identified.

## Supporting Information

S1 Table Identification of proteins sampled from microdialysates. (XLSX)

S2 Table Qualitative, quantitative and functional analysis of proteins identified from solid extraction at 12 h after wound cause. (XLSX)

S3 Table Functional cluster enrichment of defect specific proteins. (XLSX)

S4 Table Inginuity Pathway Analysis (IPA). (XLSX)

S5 Table Proteins clustering to early immune response-relevant groups. (XLSX)

S1 Text Detailed information about the methodology used for the metabolite screening. (DOCX)

## Abbreviations

BD: bone defect
CXCL1: chemokine (C-X-C motif) ligand 1
CXCL2: chemokine (C-X-C motif) ligand 2
CXCL3: chemokine (C-X-C motif) ligand 3
CXCL4: chemokine (C-X-C motif) ligand 4
CXCL7: chemokine (C-X-C motif) ligand 7
ECM: extracellular matrix
IL-6: interleukin-6
IL-10: interleukin-10
SDF-1: stromal-cell derived factor-1
STW: soft tissue wound
TGF-β1: transforming growth factor-β1

## References

[1] Sommerfeldt DW, Rubin CT. Biology of bone and how it orchestrates the form and function of the skeleton. Eur Spine J. 2001;10 Suppl 2:S86–95. 10.1007/s005860100283 11716022 PMC3611544

[2] Kolar P, Schmidt-Bleek K, Schell H, Gaber T, Toben D, Schmidmaier G, et al The early fracture hematoma and its potential role in fracture healing. Tissue Eng Part B Rev. 2010;16(4):427–34. 10.1089/ten.TEB.2009.0687 . 20196645

[3] Dimitriou R, Tsiridis E, Giannoudis PV. Current concepts of molecular aspects of bone healing. Injury. 2005;36(12):1392–404. 10.1016/j.injury.2005.07.019 . 16102764

[4] Einhorn TA. The cell and molecular biology of fracture healing. Clin Orthop Relat Res. 1998;355 (Suppl):S7–21. . 9917622 10.1097/00003086-199810001-00003

[5] Gerstenfeld LC, Cullinane DM, Barnes GL, Graves DT, Einhorn TA. Fracture healing as a post-natal developmental process: molecular, spatial, and temporal aspects of its regulation. J Cell Biochem. 2003;88(5):873–84. 10.1002/jcb.10435 . 12616527

[6] Opal SM. Phylogenetic and functional relationships between coagulation and the innate immune response. Crit Care Med. 2000;28(9 Suppl):S77–80. . 11007204 10.1097/00003246-200009001-00017

[7] Xing Z, Lu C, Hu D, Miclau T 3rd, Marcucio RS. Rejuvenation of the inflammatory system stimulates fracture repair in aged mice. J Orthop Res. 2010;28(8):1000–6. 10.1002/jor.21087 20108320 PMC2892015

[8] Broughton G 2nd, Janis JE, Attinger CE. The basic science of wound healing. Plast Reconstr Surg. 2006;117(7 Suppl):12S–34S. 10.1097/01.prs.0000225430.42531.c2 . 16799372

[9] Yager DR, Kulina RA, Gilman LA. Wound fluids: a window into the wound environment? Int J Low Extrem Wounds. 2007;6(4):262–72. 10.1177/1534734607307035 . 18048872

[10] Averbeck M, Beilharz S, Bauer M, Gebhardt C, Hartmann A, Hochleitner K, et al In situ profiling and quantification of cytokines released during ultraviolet B-induced inflammation by combining dermal microdialysis and protein microarrays. Exp Dermatol. 2006;15(6):447–54. 10.1111/j.0906-6705.2006.00429.x . 16689861

[11] Haugaa H, Thorgersen EB, Pharo A, Boberg KM, Foss A, Line PD, et al Inflammatory markers sampled by microdialysis catheters distinguish rejection from ischemia in liver grafts. Liver Transpl. 2012;18(12):1421–9. 10.1002/lt.23503 . 22767413

[12] Maurer MH, Berger C, Wolf M, Futterer CD, Feldmann RE Jr., Schwab S, et al The proteome of human brain microdialysate. Proteome Sci. 2003;1(1):7 10.1186/1477-5956-1-7 14675487 PMC317363

[13] Forster Y, Gao W, Demmrich A, Hempel U, Hofbauer LC, Rammelt S. Monitoring of the first stages of bone healing with microdialysis. Acta Orthop. 2013;84(1):76–81. 10.3109/17453674.2013.769080 23350578 PMC3584608

[14] Schenk T, Irth H, Marko-Varga G, Edholm LE, Tjaden UR, van der Greef J. Potential of on-line micro-LC immunochemical detection in the bioanalysis of cytokines. J Pharm Biomed Anal. 2001;26(5–6):975–85. . 11600310 10.1016/s0731-7085(01)00464-2

[15] Baumann S, Kalkhof S, Hackermüller J, Otto W, Tomm JM, Wissenbach DK, et al Requirements and Perspectives for Integrating Metabolomics with other Omics Data. Curr Metabolomics. 2013;1(1):13 10.2174/2213235X11301010015

[16] Bernay B, Gaillard MC, Guryca V, Emadali A, Kuhn L, Bertrand A, et al Discovering new bioactive neuropeptides in the striatum secretome using in vivo microdialysis and versatile proteomics. Mol Cell Proteomics. 2009;8(5):946–58. 10.1074/mcp.M800501-MCP200 19164277 PMC2689773

[17] Gill C, Parkinson E, Church MK, Skipp P, Scott D, White AJ, et al A qualitative and quantitative proteomic study of human microdialysate and the cutaneous response to injury. AAPS J. 2011;13(2):309–17. 10.1208/s12248-011-9269-6 21494910 PMC3085710

[18] Olausson P, Gerdle B, Ghafouri N, Larsson B, Ghafouri B. Identification of proteins from interstitium of trapezius muscle in women with chronic myalgia using microdialysis in combination with proteomics. PLoS One. 2012;7(12):e52560 10.1371/journal.pone.0052560 23300707 PMC3531451

[19] Eming SA, Koch M, Krieger A, Brachvogel B, Kreft S, Bruckner-Tuderman L, et al Differential proteomic analysis distinguishes tissue repair biomarker signatures in wound exudates obtained from normal healing and chronic wounds. J Proteome Res. 2010;9(9):4758–66. 10.1021/pr100456d . 20666496

[20] Petersen LJ, Sorensen MA, Codrea MC, Zacho HD, Bendixen E. Large pore dermal microdialysis and liquid chromatography-tandem mass spectroscopy shotgun proteomic analysis: a feasibility study. Skin Res Technol. 2013;19(4):424–31. 10.1111/srt.12063 . 23551181

[21] Kalkhof S, Forster Y, Schmidt J, Schulz MC, Baumann S, Weissflog A, et al Proteomics and metabolomics for in situ monitoring of wound healing. Biomed Res Int. 2014;2014:934848 10.1155/2014/934848 25162036 PMC4137721

[22] Cox J, Mann M. MaxQuant enables high peptide identification rates, individualized p.p.b.-range mass accuracies and proteome-wide protein quantification. Nat Biotechnol. 2008;26(12):1367–72. 10.1038/nbt.1511 . 19029910

[23] Cox J, Neuhauser N, Michalski A, Scheltema RA, Olsen JV, Mann M. Andromeda: a peptide search engine integrated into the MaxQuant environment. J Proteome Res. 2011;10(4):1794–805. 10.1021/pr101065j . 21254760

[24] Cox J, Hein MY, Luber CA, Paron I, Nagaraj N, Mann M. Accurate proteome-wide label-free quantification by delayed normalization and maximal peptide ratio extraction, termed MaxLFQ. Mol Cell Proteomics. 2014;13(9):2513–26. 10.1074/mcp.M113.031591 24942700 PMC4159666

[25] Hoeke H, Roeder S, Bertsche T, Lehmann I, Borte M, von Bergen M, et al Monitoring of drug intake during pregnancy by questionnaires and LC-MS/MS drug urine screening: evaluation of both monitoring methods. Drug Test Anal. 2015;7(8):695–702. 10.1002/dta.1767 . 25545167

[26] Tautenhahn R, Patti GJ, Rinehart D, Siuzdak G. XCMS Online: a web-based platform to process untargeted metabolomic data. Anal Chem. 2012;84(11):5035–9. 10.1021/ac300698c 22533540 PMC3703953

[27] Dennis G Jr., Sherman BT, Hosack DA, Yang J, Gao W, Lane HC, et al DAVID: Database for Annotation, Visualization, and Integrated Discovery. Genome Biol. 2003;4(5):P3 . 12734009 PMC193660

[28] Keerthikumar S, Chisanga D, Ariyaratne D, Al Saffar H, Anand S, Zhao K, et al ExoCarta: A Web-Based Compendium of Exosomal Cargo. J Mol Biol. 2015 10.1016/j.jmb.2015.09.019 . 26434508 PMC4783248

[29] Team RDC. R: A Language and Environment for Statistical Computing. Vienna, Austria: The R Foundation for Statistical Computing; 2011 Available: http://www.R-project.org/.

[30] Vu VQ. ggbiplot: A ggplot2 based biplot. 2011 Available: http://github.com/vqv/ggbiplot.

[31] Bastian O, Pillay J, Alblas J, Leenen L, Koenderman L, Blokhuis T. Systemic inflammation and fracture healing. J Leukoc Biol. 2011;89(5):669–73. 10.1189/jlb.0810446 . 21208896

[32] Franz S, Rammelt S, Scharnweber D, Simon JC. Immune responses to implants—a review of the implications for the design of immunomodulatory biomaterials. Biomaterials. 2011;32(28):6692–709. 10.1016/j.biomaterials.2011.05.078 . 21715002

[33] Marsell R, Einhorn TA. The biology of fracture healing. Injury. 2011;42(6):551–5. 10.1016/j.injury.2011.03.031 21489527 PMC3105171

[34] Kirbs C, Kloft C. In vitro microdialysis recovery and delivery investigation of cytokines as prerequisite for potential biomarker profiling. Eur J Pharm Sci. 2014;57:48–59. 10.1016/j.ejps.2013.11.006 . 24246312

[35] Perl M, Gebhard F, Knoferl MW, Bachem M, Gross HJ, Kinzl L, et al The pattern of preformed cytokines in tissues frequently affected by blunt trauma. Shock. 2003;19(4):299–304. . 12688538 10.1097/00024382-200304000-00001

[36] Clausen TS, Kaastrup P, Stallknecht B. Proinflammatory tissue response and recovery of adipokines during 4 days of subcutaneous large-pore microdialysis. J Pharmacol Toxicol Methods. 2009;60(3):281–7. 10.1016/j.vascn.2009.03.001 . 19328242

[37] Schutte RJ, Oshodi SA, Reichert WM. In vitro characterization of microdialysis sampling of macromolecules. Anal Chem. 2004;76(20):6058–63. 10.1021/ac0493626 . 15481954

[38] Plock N, Kloft C. Microdialysis—theoretical background and recent implementation in applied life-sciences. Eur J Pharm Sci. 2005;25(1):1–24. 10.1016/j.ejps.2005.01.017 . 15854796

[39] Waelgaard L, Pharo A, Tonnessen TI, Mollnes TE. Microdialysis for monitoring inflammation: efficient recovery of cytokines and anaphylotoxins provided optimal catheter pore size and fluid velocity conditions. Scand J Immunol. 2006;64(3):345–52. 10.1111/j.1365-3083.2006.01826.x . 16918704

[40] Ao X, Stenken JA. Microdialysis sampling of cytokines. Methods. 2006;38(4):331–41. 10.1016/j.ymeth.2005.11.012 . 16487724

[41] Heinrich PC, Castell JV, Andus T. Interleukin-6 and the acute phase response. Biochem J. 1990;265(3):621–36. 1689567 10.1042/bj2650621 PMC1133681

[42] Nakagawa H, Ando Y, Takano K, Sunada Y. Differential production of chemokines and their role in neutrophil infiltration in rat allergic inflammation. Int Arch Allergy Immunol. 1998;115(2):137–43. . 9482702 10.1159/000023893

[43] Nakagawa H, Komorita N, Shibata F, Ikesue A, Konishi K, Fujioka M, et al Identification of cytokine-induced neutrophil chemoattractants (CINC), rat GRO/CINC-2 alpha and CINC-2 beta, produced by granulation tissue in culture: purification, complete amino acid sequences and characterization. Biochem J. 1994;301 (Pt 2):545–50. 8043001 10.1042/bj3010545 PMC1137115

[44] Shibata F, Konishi K, Kato H, Komorita N, al-Mokdad M, Fujioka M, et al Recombinant production and biological properties of rat cytokine-induced neutrophil chemoattractants, GRO/CINC-2 alpha, CINC-2 beta and CINC-3. Eur J Biochem. 1995;231(2):306–11. . 7635142 10.1111/j.1432-1033.1995.tb20701.x

[45] Takano K, Nakagawa H. Contribution of cytokine-induced neutrophil chemoattractant CINC-2 and CINC-3 to neutrophil recruitment in lipopolysaccharide-induced inflammation in rats. Inflamm Res. 2001;50(10):503–8. . 11713904 10.1007/PL00000226

[46] Gleissner CA, von Hundelshausen P, Ley K. Platelet chemokines in vascular disease. Arterioscler Thromb Vasc Biol. 2008;28(11):1920–7. 10.1161/ATVBAHA.108.169417 18723831 PMC2657037

[47] Hagiwara H, Mitsumata M, Yamane T, Jin X, Yoshida Y. Laminar shear stress-induced GRO mRNA and protein expression in endothelial cells. Circulation. 1998;98(23):2584–90. . 9843467 10.1161/01.cir.98.23.2584

[48] Rossi D, Zlotnik A. The biology of chemokines and their receptors. Annu Rev Immunol. 2000;18:217–42. 10.1146/annurev.immunol.18.1.217 . 10837058

[49] Eash KJ, Greenbaum AM, Gopalan PK, Link DC. CXCR2 and CXCR4 antagonistically regulate neutrophil trafficking from murine bone marrow. J Clin Invest. 2010;120(7):2423–31. 10.1172/JCI41649 20516641 PMC2898597

[50] Kohler A, De Filippo K, Hasenberg M, van den Brandt C, Nye E, Hosking MP, et al G-CSF-mediated thrombopoietin release triggers neutrophil motility and mobilization from bone marrow via induction of Cxcr2 ligands. Blood. 2011;117(16):4349–57. 10.1182/blood-2010-09-308387 21224471 PMC3087483

[51] Sokol CL, Luster AD. The chemokine system in innate immunity. Cold Spring Harb Perspect Biol. 2015;7(5). 10.1101/cshperspect.a016303 . 25635046 PMC4448619

[52] Gauglitz GG, Song J, Herndon DN, Finnerty CC, Boehning D, Barral JM, et al Characterization of the inflammatory response during acute and post-acute phases after severe burn. Shock. 2008;30(5):503–7. 10.1097/SHK.0b013e31816e3373 . 18391855 PMC7863568

[53] Brandt E, Petersen F, Ludwig A, Ehlert JE, Bock L, Flad HD. The beta-thromboglobulins and platelet factor 4: blood platelet-derived CXC chemokines with divergent roles in early neutrophil regulation. J Leukoc Biol. 2000;67(4):471–8. . 10770278 10.1002/jlb.67.4.471

[54] Vasicek TW, Jackson MR, Poseno TM, Stenken JA. In vivo microdialysis sampling of cytokines from rat hippocampus: comparison of cannula implantation procedures. ACS Chem Neurosci. 2013;4(5):737–46. 10.1021/cn400025m 23480171 PMC3656755

[55] Barrientos S, Stojadinovic O, Golinko MS, Brem H, Tomic-Canic M. Growth factors and cytokines in wound healing. Wound Repair Regen. 2008;16(5):585–601. 10.1111/j.1524-475X.2008.00410.x . 19128254

[56] Kitaori T, Ito H, Schwarz EM, Tsutsumi R, Yoshitomi H, Oishi S, et al Stromal cell-derived factor 1/CXCR4 signaling is critical for the recruitment of mesenchymal stem cells to the fracture site during skeletal repair in a mouse model. Arthritis Rheum. 2009;60(3):813–23. 10.1002/art.24330 . 19248097

[57] Park JE, Barbul A. Understanding the role of immune regulation in wound healing. Am J Surg. 2004;187(5A):11S–6S. 10.1016/S0002-9610(03)00296-4 . 15147986

[58] Witte MB, Barbul A. General principles of wound healing. Surg Clin North Am. 1997;77(3):509–28. . 9194878 10.1016/s0039-6109(05)70566-1

[59] Clark RA, Lanigan JM, DellaPelle P, Manseau E, Dvorak HF, Colvin RB. Fibronectin and fibrin provide a provisional matrix for epidermal cell migration during wound reepithelialization. J Invest Dermatol. 1982;79(5):264–9. . 6752288 10.1111/1523-1747.ep12500075

[60] Drew AF, Liu H, Davidson JM, Daugherty CC, Degen JL. Wound-healing defects in mice lacking fibrinogen. Blood. 2001;97(12):3691–8. . 11389004 10.1182/blood.v97.12.3691

[61] Lebreton JP, Joisel F, Raoult JP, Lannuzel B, Rogez JP, Humbert G. Serum concentration of human alpha 2 HS glycoprotein during the inflammatory process: evidence that alpha 2 HS glycoprotein is a negative acute-phase reactant. J Clin Invest. 1979;64(4):1118–29. 10.1172/JCI109551 90057 PMC372224

[62] Nieminen J, St-Pierre C, Sato S. Galectin-3 interacts with naive and primed neutrophils, inducing innate immune responses. J Leukoc Biol. 2005;78(5):1127–35. 10.1189/jlb.1204702 . 16260586

[63] Sano H, Hsu DK, Yu L, Apgar JR, Kuwabara I, Yamanaka T, et al Human galectin-3 is a novel chemoattractant for monocytes and macrophages. J Immunol. 2000;165(4):2156–64. . 10925302 10.4049/jimmunol.165.4.2156

[64] Clemmensen SN, Bohr CT, Rorvig S, Glenthoj A, Mora-Jensen H, Cramer EP, et al Olfactomedin 4 defines a subset of human neutrophils. J Leukoc Biol. 2012;91(3):495–500. 10.1189/jlb.0811417 22187488 PMC3289394

[65] Welin A, Amirbeagi F, Christenson K, Bjorkman L, Bjornsdottir H, Forsman H, et al The human neutrophil subsets defined by the presence or absence of OLFM4 both transmigrate into tissue in vivo and give rise to distinct NETs in vitro. PLoS One. 2013;8(7):e69575 10.1371/journal.pone.0069575 23922742 PMC3726694

[66] Liu W, Yan M, Liu Y, McLeish KR, Coleman WG Jr., Rodgers GP. Olfactomedin 4 inhibits cathepsin C-mediated protease activities, thereby modulating neutrophil killing of Staphylococcus aureus and Escherichia coli in mice. J Immunol. 2012;189(5):2460–7. 10.4049/jimmunol.1103179 22844115 PMC3424379

[67] Wang CY, Lin CF. Annexin A2: its molecular regulation and cellular expression in cancer development. Dis Markers. 2014;2014:308976 10.1155/2014/308976 24591759 PMC3925611

[68] Swisher JF, Burton N, Bacot SM, Vogel SN, Feldman GM. Annexin A2 tetramer activates human and murine macrophages through TLR4. Blood. 2010;115(3):549–58. 10.1182/blood-2009-06-226944 19965653 PMC2810992

[69] Swisher JF, Khatri U, Feldman GM. Annexin A2 is a soluble mediator of macrophage activation. J Leukoc Biol. 2007;82(5):1174–84. 10.1189/jlb.0307154 . 17715360

[70] Ryckman C, Vandal K, Rouleau P, Talbot M, Tessier PA. Proinflammatory activities of S100: proteins S100A8, S100A9, and S100A8/A9 induce neutrophil chemotaxis and adhesion. J Immunol. 2003;170(6):3233–42. . 12626582 10.4049/jimmunol.170.6.3233

[71] Xu J, Gao XP, Ramchandran R, Zhao YY, Vogel SM, Malik AB. Nonmuscle myosin light-chain kinase mediates neutrophil transmigration in sepsis-induced lung inflammation by activating beta2 integrins. Nat Immunol. 2008;9(8):880–6. 10.1038/ni.1628 18587400 PMC2553242

[72] Wilgus TA, Roy S, McDaniel JC. Neutrophils and Wound Repair: Positive Actions and Negative Reactions. Adv Wound Care (New Rochelle). 2013;2(7):379–88. 10.1089/wound.2012.0383 24527354 PMC3763227

[73] Foger N, Rangell L, Danilenko DM, Chan AC. Requirement for coronin 1 in T lymphocyte trafficking and cellular homeostasis. Science. 2006;313(5788):839–42. 10.1126/science.1130563 . 16902139

[74] Wabnitz GH, Kocher T, Lohneis P, Stober C, Konstandin MH, Funk B, et al Costimulation induced phosphorylation of L-plastin facilitates surface transport of the T cell activation molecules CD69 and CD25. Eur J Immunol. 2007;37(3):649–62. 10.1002/eji.200636320 . 17294403

[75] Blume KE, Soeroes S, Waibel M, Keppeler H, Wesselborg S, Herrmann M, et al Cell surface externalization of annexin A1 as a failsafe mechanism preventing inflammatory responses during secondary necrosis. J Immunol. 2009;183(12):8138–47. 10.4049/jimmunol.0902250 . 20007579

[76] Pangburn MK, Schreiber RD, Muller-Eberhard HJ. Human complement C3b inactivator: isolation, characterization, and demonstration of an absolute requirement for the serum protein beta1H for cleavage of C3b and C4b in solution. J Exp Med. 1977;146(1):257–70. 301546 10.1084/jem.146.1.257 PMC2180748

[77] Eming SA, Krieg T, Davidson JM. Inflammation in wound repair: molecular and cellular mechanisms. J Invest Dermatol. 2007;127(3):514–25. 10.1038/sj.jid.5700701 . 17299434

[78] Grinnell F, Zhu M. Fibronectin degradation in chronic wounds depends on the relative levels of elastase, alpha1-proteinase inhibitor, and alpha2-macroglobulin. J Invest Dermatol. 1996;106(2):335–41. . 8601737 10.1111/1523-1747.ep12342990

[79] Kuckleburg CJ, Tilkens SB, Santoso S, Newman PJ. Proteinase 3 contributes to transendothelial migration of NB1-positive neutrophils. J Immunol. 2012;188(5):2419–26. 10.4049/jimmunol.1102540 22266279 PMC3288489

[80] Pham CT. Neutrophil serine proteases: specific regulators of inflammation. Nat Rev Immunol. 2006;6(7):541–50. 10.1038/nri1841 . 16799473

[81] Romer J, Bugge TH, Pyke C, Lund LR, Flick MJ, Degen JL, et al Impaired wound healing in mice with a disrupted plasminogen gene. Nat Med. 1996;2(3):287–92. . 8612226 10.1038/nm0396-287

[82] Silverman GA, Bird PI, Carrell RW, Church FC, Coughlin PB, Gettins PG, et al The serpins are an expanding superfamily of structurally similar but functionally diverse proteins. Evolution, mechanism of inhibition, novel functions, and a revised nomenclature. J Biol Chem. 2001;276(36):33293–6. 10.1074/jbc.R100016200 . 11435447

[83] Bost F, Diarra-Mehrpour M, Martin J-P. Inter-α-trypsin inhibitor proteoglycan family. European Journal of Biochemistry. 1998;252(3):339–46. 10.1046/j.1432-1327.1998.2520339.x 9546647

[84] Sugimori T, Cooley J, Hoidal JR, Remold-O'Donnell E. Inhibitory properties of recombinant human monocyte/neutrophil elastase inhibitor. Am J Respir Cell Mol Biol. 1995;13(3):314–22. 10.1165/ajrcmb.13.3.7654387 . 7654387

[85] Braciak TA, Northemann W, Hudson GO, Shiels BR, Gehring MR, Fey GH. Sequence and acute phase regulation of rat alpha 1-inhibitor III messenger RNA. J Biol Chem. 1988;263(8):3999–4012. . 2831216

[86] Lonberg-Holm K, Reed DL, Roberts RC, Hebert RR, Hillman MC, Kutney RM. Three high molecular weight protease inhibitors of rat plasma. Isolation, characterization, and acute phase changes. J Biol Chem. 1987;262(1):438–45. . 2432067

[87] Yang LC, Marsala M, Yaksh TL. Characterization of time course of spinal amino acids, citrulline and PGE2 release after carrageenan/kaolin-induced knee joint inflammation: a chronic microdialysis study. Pain. 1996;67(2–3):345–54. . 8951928 10.1016/0304-3959(96)03106-5

[88] Famularo G, De Simone C, Trinchieri V, Mosca L. Carnitines and its congeners: a metabolic pathway to the regulation of immune response and inflammation. Ann N Y Acad Sci. 2004;1033:132–8. 10.1196/annals.1320.012 . 15591010

