# Supplementary material for: Microdialysis Sampling from Wound Fluids Enables Quantitative Assessment of Cytokines, Proteins, and Metabolites Reveals Bone Defect-Specific Molecular Profiles
Source: PLoS One. 2016 Jul 21;11(7):e0159580. doi: 10.1371/journal.pone.0159580 (PMC4956113; doi:10.1371/journal.pone.0159580)
Supplement: S1 Text — (DOCX) [file pone.0159580.s006.docx]

# LC-MS Analysis for Metabolomic Profiling

For untargeted Metabolomics Profiling a Waters Acquity (Waters, Eschborn, Germany) Ultra High Performance Liquid Chromatography (UHPLC) system coupled to an Orbitrap Velos (Thermo Fisher, TF, Dreieich, Germany) mass analyser was used. Gradient elution was performed on a Waters Acquity CSH C18 (100×2.1 mm, 1.7μm) column at a flow rate (FR) of 0.5 ml/min. The mobile phase consisted of 10 mM aqueous ammonium formate containing 0.1 % formic acid (pH 3.4, eluent A) and acetonitrile containing 0.1 % formic acid (eluent B). The gradient was programmed as follows: 0-1.0 min 98 % A, 1.0‑3.0 min to 90 % A, 3.0-5.0 min to 85 % A, 5.0-7.5 min to 80 % A, 7.5-10.0 min to 75 % A, 10.0-11.5 min to 70 % A, 11.5-13.0 min to 65 % A, 13.0-14.5 min to 50 % A, 14.5-16.0 min to 40 % A, 16.0-19.0 min to 0 % A, 19.0-21.0 min hold 0 % A, 21.0-22.0 min to 98 % A, 22.0-25.0 min hold 98 % A. The injection volume was 10 μl. After each injection a needle wash was conducted using 1.0 ml methanol/water (85:15, v:v) solution. Additionally, a “blank” sample containing a methanol/water (85:15, v:v) solution was analysed and evaluated particularly with regard to carry over after each authentic urine sample.

The MS conditions were as follows: positive ionization mode using a heated ESI source (HESI II, TF, Dreieich, Germany) sheath gas, nitrogen at FR of 30 arbitrary units (AU); auxiliary gas, nitrogen at FR of 10 AU; sweep gas at FR of 7 AU; vaporizer temperature, 250 °C; source voltage, 3.00 kV; ion transfer capillary temperature, 300 °C; capillary voltage, 31 V; and S-lens RF level, 88 %. Automatic gain control was set to 1.000.000 ions for full scan and 10.000 ions for MS^n^. The maximum injection time for full scan (MS^1^ stage) was set to 100 ms. Collision‑induced dissociation (CID)-MS^n^ experiments were performed on precursor ions selected from MS^1^ using data-dependent acquisition (DDA): MS^1^ was performed in the full scan mode using high resolution (m/z 100–800; resolution: 15.000). MS^2^ and MS^3^ were performed in the DDA mode using the nominal mass ion trap mass analyser: four DDA MS^2^ scan filters were chosen to provide MS^2^ on the four most intense signals from MS^1^, and additionally, eight MS^3^ scan filters were chosen to record MS^3^ on the most and second most intense signals from the MS^2^. Normalized wideband collision energies were 35.0 % for MS^2^ and 40.0 % for MS^3^. Other settings were as follows for MS^2^: minimum signal threshold, 250 counts; isolation width, 1.5 u; for MS^3^: minimum signal threshold, 100 counts; isolation width, 2.0 u; dynamic exclusion mode: repeat counts, 2; repeat duration, 30 s; exclusion list, 500; exclusion duration, 30 s. Activation Q of 0.25 and an activation time of 18 ms were applied, after optimization to a calibration point as described by Wissenbach et al. (Wissenbach *et al.*, 2011).

# Data Evaluation for Metabolomic Profiling

Data analysis was performed by XCMS online (Tautenhahn *et al.*, 2012). In detail feature detection was carried out by “centWave” method, using 3 ppm maximal tolerated m/z deviation in consecutive scans, 10 and 60 second minimum and maximum peak width. Retention time correction was achieved by oriwarp method using profStep 1 setting. Features were aligned with the following settings: bw = 5; mzwid = 0.015, and minfrac = 0.5. Statistical evaluation was performed by unpaired parametric t-test (welch t-test) with a p-value threshold of 0.05 and a fold-change threshold of 1.5. For annotation “isotope” method was used (ppm = 5 and absolute m/z error = 0.015. Identification was achieved for “M+H” with a 5 ppm tolerance using “human mfn” as matrix model.

## References

Tautenhahn R, Patti GJ, Rinehart D, Siuzdak G 2012, XCMS Online: a web-based platform to process untargeted metabolomic data, *Anal Chem*, **84**: 5035-9

Wissenbach DK, Meyer MR, Remane D, Weber AA, Maurer HH 2011, Development of the first metabolite-based LC-MS(n) urine drug screening procedure-exemplified for antidepressants, *Anal Bioanal Chem*, **400**: 79-88
